# A history of male migration in and out of the Green Sahara

**DOI:** 10.1186/s13059-018-1410-8

**Published:** 2018-03-13

**Authors:** Yali Xue

**Affiliations:** Wellcome Sanger Institute, Wellcome Genome Campus, Hinxton, Cambridgeshire, CB10 1SA UK

## Abstract

The Sahara was once fertile; a recent study identifies human Y-chromosomal lineages that flourished in this Green Sahara and their relation to present-day populations.

## Introduction

Modern humans originated in Africa at least 300 thousand years ago (kya). The migration out of Africa (~ 60 kya) was central to human history, but the subsequent migrations and demographic events within Africa, stimulated by climate changes, developments in technology and other historical factors remain under-appreciated and under-studied. A recent study [1], focusing on the consequences of climatic changes in the Saharan region over the past 12 thousand years, fills one of the gaps in our knowledge of human migration.

## Y-chromosomal lineages from the Green Sahara

The transformation of the Sahara over the past 12 thousand years has been profound: a once hospitable environment with lakes, savannahs and animals 5–12 kya (referred to as the Green Sahara or the Holocene Wet Phase) has become a hostile environment that forms the largest non-Polar desert on earth. This transition is expected to have had major consequences for the human inhabitants of the region and may have left imprints in the genomes of nearby present-day populations, but can these traces be identified?

D’Atanasio et al. [1] focused on the Y chromosome to address this question. The ~ 60 Mb Y chromosome is inherited from father to son, and no recombination occurs on most of its length. Therefore, this chromosome gradually accumulates mutations and thus provides a particularly simple and detailed record of human history, at least as it has impacted men. This record is enhanced by the historical tendency of men to remain near their birthplace, leading to a strong geographical structure of Y chromosome types. For decades, dedicated researchers have distinguished Y-chromosomal lineages using genetic variants (identifying ‘haplogroups’) and have established the geographical distributions of these variants [2]. In the past few years, advances in genomic technology have made large-scale sequencing of Y chromosomes feasible, which in turn has transformed the field by enabling measurement of the mutation rate and construction of detailed, well-calibrated phylogenies. It is thus possible to use the distributions of present-day Y-chromosomal lineages to infer the historical movements of men.

**Fig. 1:**
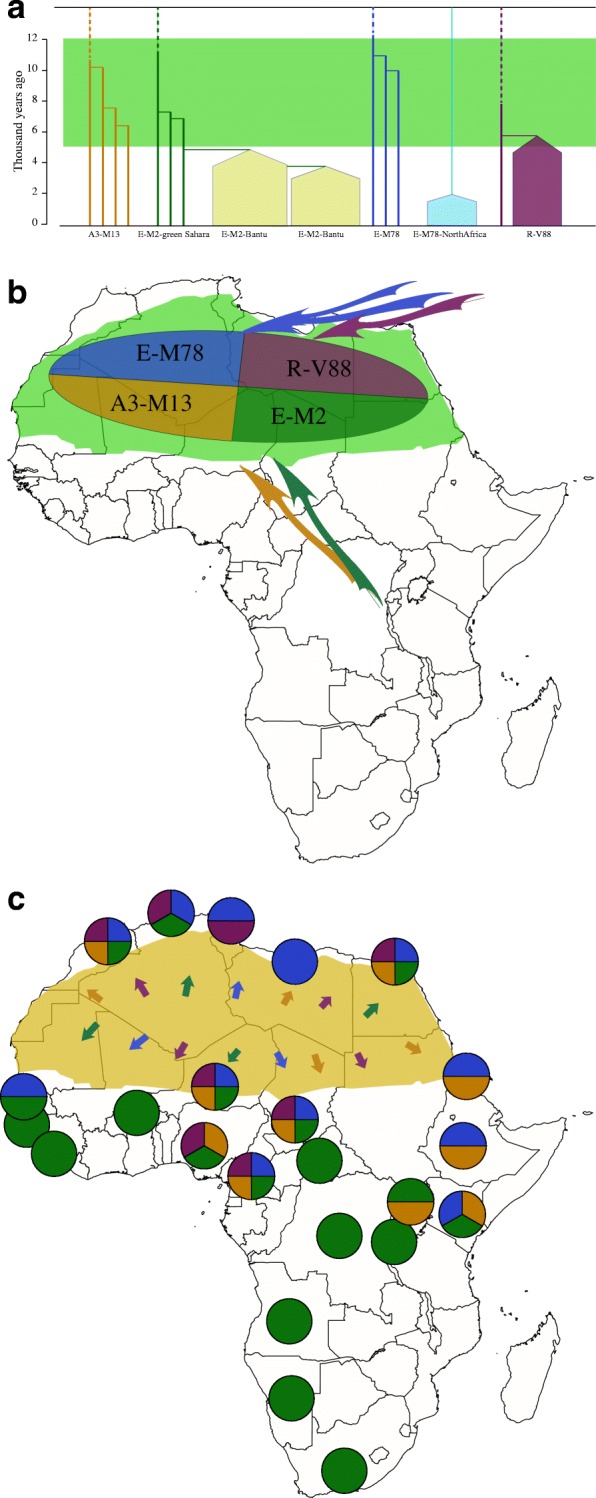
Simplified Y-chromosomal phylogeny and inferred past or observed present-day distribution of relevant Y-chromosomal lineages. **a** Calibrated phylogenetic tree of Y-chromosomal lineages discussed in the text. *Green shading* represents the period when the present-day Sahara Desert was green and fertile. Lineages represented by *filled pentagons* have undergone very rapid expansions. **b** The Green Sahara period 5–12 kya. *Green shading* indicates that the present-day Sahara Desert was green and fertile. The colors within the *large oval* represent the four Y-chromosomal haplogroups deduced to be present in the region at this time; specific locations are not implied. The *arrows* indicate the inferred origins of these haplogroups to the north or south, but specific origins and routes are not implied. **c** The present-day distributions of the four Green Saharan Y-chromosomal haplogroups. *Yellow shading* indicates the Sahara Desert. Each *circle* represents a sampled population, with the presence or absence of the four Green Saharan haplogroups shown by the colored sectors; other haplogroups may also be present in these populations, but are not shown. The *small arrows* indicate the inferred northwards and southwards movements of these haplogroups when the Sahara became uninhabitable. Figure based on data from [1]

D’Atanasio et al. [1] hypothesized that, because the Sahara has been such a strong barrier to human migration for the past 5000 years, haplogroups that are found in the current populations both to the north and to the south of the Sahara must have spread during the green phase. Previous work had identified four such haplogroups: A3-M13, E-M2, E-M78 and R-V88. (The letter prefix specifies the major Y lineage, and the suffix the particular Y-SNP that defines the lineage.) Targeted sequencing of the Y chromosomes (~ 3.3 Mb) of a set of 104 men, most of whom carried one of these four lineages, allowed D’Atanasio and colleagues to construct a dated phylogenetic tree. This tree, shown in a simplified form in Fig. 1a, revealed the coalescent time of these haplogroups: that is, the period of time that has elapsed since the most recent common ancestor of the haplogroups lived. These time points mostly lay within the 5–12 kya green period, although E-M78 showed a more complex history with some older and more diverse lineages. So, for example, all of the men carrying the A3-M13 Y chromosome are descendants down the male lineage of a single ancestor who lived 10.73 (SD 0.91) kya. Although there is no direct evidence that this ancestor lived in the Green Sahara, it is a simple and reasonable inference (Fig. 1b). Notably, the R-V88 lineage shows an additional striking feature: a very rapid expansion (visualized as lineages in the phylogeny branching so closely together that no mutations occurred between the origin of the different branches) at 5.73 (SD 0.49) kya.

The authors extended their findings by genotyping 142 informative SNPs (many newly discovered in this study) in 7955 additional men from 145 African, Eurasian and African-American populations. This enabled them to better understand both the likely origins of the four haplogroups earlier than 12 kya (Fig. 1b) and the locations of the present-day descendants (Fig. 1c). E-M78 and R-V88 [3] probably originated from the north because the most closely related lineages were found there, and A-M13 and E-M2 from the south.

There are some caveats to the conclusions; for example, the authors used their own estimate of the mutation rate, which happened to be slightly slower than the commonly used one, leading to a slight difference in time estimations that need to be taken into account when comparing with other estimates. Nevertheless, the lineage distributions and their fit with the time of the Green Sahara are very robust. How do these conclusions fit within a broader context?

## Insights from the Green Saharan Y-chromosomal findings

It is widely accepted that sub-Saharan Y chromosomes are dominated by E-M2 lineages carried by Bantu-speaking farmers as they expanded from West Africa starting < 5 kya, reaching South Africa within recent centuries [4]. The E-M2-Bantu lineages lie phylogenetically within the E-M2-Green Sahara lineage and show at least three explosive lineage expansions beginning 4.9–5.3 kya [5] (Fig. 1a). These events of E-M2-Bantu expansion are slightly later than the R-V88 expansion, and highlight the range of male demographic changes in the mid-Holocene. North of the Sahara, in addition to the four trans-Saharan haplogroups, haplogroup E-M81 (which diverged from E-M78 ~ 13 kya) became very common in present-day populations as a result of another massive expansion ~ 2 kya [6] (Fig. 1a).

Although Y chromosomes exist within populations and so share and reflect the general history of those populations, they can sometimes show some departures from other parts of the genome that result from differences in male and female behaviors. D’Atanasio et al. [1] highlight one such contrast in their study. Present-day North African populations show substantial sub-Saharan autosomal and mtDNA genetic components ascribed to the Roman and Arab slave trades 1–2 kya [7], but carry few sub-Saharan Y lineages from this source, probably reflecting the smaller numbers of male slaves and their reduced reproductive opportunities when compared to those of female slaves. The sub-Saharan Y chromosomes in these North African populations thus originate predominantly from the earlier Green Sahara period.

In this part of Africa, the indigenous languages that are spoken belong to three of the four African linguistic families (Afro-Asiatic, Nilo-Saharan and Niger-Congo). Interestingly, these languages show non-random associations with Y lineages. For example, Chadic languages within the Afro-Asiatic family are associated with haplogroup R-V88, whereas Nilo-Saharan languages are associated with specific sublineages within A3-M13 and E-M78, further illustrating the complex human history of the region.

## Perspectives

Ancient DNA studies usually show that the human past was much more complicated than can be inferred from studies of the DNA in present-day populations [8]. With the demonstration of successful recovery and analysis of ancient African DNA as old as ~ 8 kya from Malawi [9] and > 2 kya from Egypt [10], it seems that ancient DNA may be recoverable more widely from Africa, and thus could inform us more directly about Y-chromosomal history and population movements during the Green Sahara period. The current study raises many questions that remain to be addressed. For example, what are the reasons for the very rapid R-V88 expansion 5–6 kya [1] and E-M81 expansion ~ 2 kya [6], and how do these expansions fit within general worldwide patterns of male-specific expansions, which in other cases have been linked to cultural and technological changes [5]?

## Abbreviation

kya: Thousand years ago
